# Pharmacokinetics of Intravitreal Anti-VEGF Drugs in Age-Related Macular Degeneration

**DOI:** 10.3390/pharmaceutics11080365

**Published:** 2019-07-31

**Authors:** Laura García-Quintanilla, Andrea Luaces-Rodríguez, María Gil-Martínez, Cristina Mondelo-García, Olalla Maroñas, Víctor Mangas-Sanjuan, Miguel González-Barcia, Irene Zarra-Ferro, Pablo Aguiar, Francisco J. Otero-Espinar, Anxo Fernández-Ferreiro

**Affiliations:** 1 Pharmacy Department, University Clinical Hospital of Santiago de Compostela (SERGAS), 15706 Santiago de Compostela, Spain; 2 Pharmacology Group, Health Research Institute of Santiago de Compostela (FIDIS), 15706 Santiago de Compostela, Spain; 3 Department of Pharmacology, Pharmacy and Pharmaceutical Technology, Faculty of Pharmacy, University of Santiago de Compostela (USC), 15782 Santiago de Compostela, Spain; 4 Ophthalmology Department, University Clinical Hospital of Santiago de Compostela (SERGAS), 15706 Santiago de Compostela, Spain; 5 Genomic Medicine Group, Galician Public Foundation of Genomic Medicine, Health Research Institute of Santiago de Compostela (FIDIS), 15706 Santiago de Compostela, Spain; 6 Department of Pharmacy and Pharmaceutical Technology and Parasitology, University of Valencia, 46100 Valencia, Spain; 7 Interuniversity Research Institute for Molecular Recognition and Technological Development, Polytechnic University of Valencia, 46100 Valencia, Spain; 8 Nuclear Medicine Department, University Clinical Hospital of Santiago de Compostela (SERGAS), 15706 Santiago de Compostela, Spain; 9 Molecular Imaging Group, Health Research Institute of Santiago de Compostela (FIDIS), 15706 Santiago de Compostela, Spain

**Keywords:** vascular endothelial growth factor/antagonists & inhibitors, ranibizumab, aflibercept, bevacizumab, Age-Related Macular Degeneration, pharmacokinetics, intravitreal

## Abstract

Intravitreal administration of anti-vascular endothelial growth factor (VEGF) antibodies has become the standard treatment for Age-Related Macular Degeneration; however, the knowledge of their pharmacokinetics is limited. A comprehensive review of the preclinical and clinical pharmacokinetic data that were obtained in different studies with intravitreal bevacizumab, ranibizumab, and aflibercept has been conducted. Moreover, the factors that can influence the vitreous pharmacokinetics of these drugs, as well as the methods that were used in the studies for analytical determination, have been exposed. These anti-VEGF drugs present different charge and molecular weights, which play an important role in vitreous distribution and elimination. The pharmacokinetic parameters that were collected differ depending on the species that were involved in the studies and on physiological and pathological conditions, such as vitrectomy and lensectomy. Knowledge of the intravitreal pharmacokinetics of the anti-VEGF drugs that were used in clinical practice is of vital importance.

## 1. Introduction

Age-Related Macular Degeneration (AMD) is the leading cause of irreversible visual impairment among individuals over the age of 65 years all around the world (between 30 and 50 million people). It is expected that its prevalence will double in the next few decades, and it is estimated that 280 million people will be affected by 2040 [1,2].

The disease almost always begins as a non-neovascular form of AMD and it may progress to the neovascular form in one or both eyes [3]. A progressive deterioration in the macula characterises the non-neovascular form, which causes central vision loss. The neovascular form is caused by the abnormal development of blood vessels under the macula, leading to the leakage of fluid and blood causing inflammation. The latter form progresses more rapidly, and it can cause severe vision loss within a few months if left untreated [4]. 

The cause of the disease is multifactorial (i.e. age, ethnic origin and a combination of genetic and environmental factors) [5]. Several treatments for neovascular AMD have been widely studied, such as laser photocoagulation and photodynamic eye therapy with verteporfin, but nowadays the standard treatment consists of intravitreal injections of inhibitors of vascular endothelial growth factor (anti-VEGF). The anti-VEGF monoclonal antibodies that were used to treat AMD include the approved intravitreal administration of pegaptanib, ranibizumab, and aflibercept and the off-label intravitreal administration of bevacizumab and Ziv-aflibercept [6,7]. 

Nowadays, in clinical practice, it is very difficult to achieve adequate therapeutic drug levels in the vitreous humour through topical ocular or systemic administration, which is mainly due to the existence of physiological barriers. Oral treatments could be an attractive treatment option; however, they have failed to show benefits in combination with intravitreal anti-VEGF treatments and they are still being evaluated in monotherapy [8]. Therefore, intravitreal injections are still the most appropriate method for treating pathologies that affect the posterior segment of the eye [9]. The frequency of administration of anti-VEGF drugs plays a key role, as their administration is currently not standardised in clinical practice and therefore different administration schedules coexist. Fixed regimens were evaluated in pivotal studies [10,11,12], in which the patients received monthly or bimonthly injections on a continuous basis over the follow up months [10,11,13]. Fixed monthly injections offer the best visual outcome, but this regimen is not commonly followed outside clinical trials due to the increased number of required visits to the ophthalmologist [14]. In addition, the followed regimen can have significant economic repercussions due to the high cost of these treatments [15]. The two most commonly followed treatment regimens are the pro re nata (PRN), which consists of treating if reactivation, and the Treat and Extend (T&E) strategy. The latter consists of a proactive treatment regimen where the key is to treat the patient before the disease activity appears. It was created to reduce the frequency of injections and it is the most accepted treatment regimen [16,17]. T&E consists of a loading phase of three monthly injections, followed by a progressive lengthening of the treatment intervals by one or two weeks as long as no activity is detected [18]. If disease activity is detected during any visit, treatment intervals are reduced to the interval used prior to the extension. A recent meta-analysis has shown that the T&E regime has a mean of 6.9 fewer injections at 24 months when compared to monthly injections yielding similar visual acuity results. Moreover, when compared to the PRN strategy, T&E has revealed an improvement of 6.18 more letters than PRN in terms of visual gains, however a mean of 1.44 more injections was required for the T&E as compared to PRN regimen at 12 months [16].

Normally, the frequency of administration should be based on the half-life of the drug (*t*_1/2_) in order to achieve a sustained therapeutic drug concentration in the vitreous. Direct determination of the vitreous drug levels requires invasive techniques, and for this reason, these type of studies are limited to the preclinical field [19,20]. Therefore, most clinical pharmacokinetics studies rely on indirect blood measurements, which have been mainly restricted to pivotal studies, which have not been studied in the great majority of post-authorisation trials [21,22]. For these reasons, the information that is available in this field is very limited.

The present review collects the most relevant aspects of the intravitreal pharmacokinetics of anti-VEGF drugs in AMD. For that purpose, an extensive review of the preclinical and clinical pharmacokinetic studies that have been published in this field was carried out. Moreover, information regarding the factors involved in the vitreous distribution and clearance, the methods for the quantification of anti-VEGF antibodies, and the utility of population models have also been compiled.

## 2. Pharmacokinetics of Anti-VEFG Drugs

In is very difficult to perform intravitreal pharmacokinetic studies on humans, given that taking vitreous samples is an invasive procedure; therefore, most of the studies have focused on preclinical research. 

### 2.1. Ranibizumab

Ranibizumab is a fragment of a monoclonal antibody that does not contain the Fc region (heavy chains) and with affinity for all subtypes of VEGF-A (Table 1). It has been approved for the treatment of neovascular AMD based on the results of two clinical trials (ANCHOR and MARINE), where 0.5 mg was administered on a monthly regimen observing improvements in visual acuity with gains of 11.3 letters in the ANCHOR trial and 7.2 letters in the MARINA trial, as compared to the control groups [10,11].

#### 2.1.1. Animal Studies

In a pharmacokinetic analysis that was performed by Bakri et al., 0.5 mg of ranibizumab was intravitreally injected in the right eye of male Dutch-belted rabbits. While using a non-compartment model, they determined that the half-life of ranibizumab was 2.88 days in the vitreous humour and 2.84 days in the aqueous humour. The mean resident time (MRT) was 4 and 6.8 days in vitreous and aqueous humour, respectively, and no serum concentrations were detected [21].

Other studies have found vitreous half-lives within the same range. A study using I-124 labelled ranibizumab in rabbits and quantified by PET/CT obtained a similar vitreous half-life (2.81 days), but a two-compartment model was applied in this case [26]. Another study calculated a vitreous half-life of 2.75 days with a one-compartment model [27]. In all the aforementioned studies of the pharmacokinetics of ranibizumab in rabbits, the standard dose of ranibizumab (0.5 mg/0.05 mL) was injected in just one rabbit eye (unilateral injection). However, the authors of the following study decided to perform a bilateral injection, with a different dose to the standard. They injected 0.625 mg/0.05 mL in both eyes, obtaining a vitreous half-life of 2.9 days with a one-compartment model [28], which is comparable to the half-life that was calculated in the other conditions.

Other studies have tested ranibizumab pharmacokinetics in monkeys as a non-human primate model. Christoforidis et al. performed a study with owl monkeys with I-124 radiolabelled ranibizumab in the same way as they performed the study in rabbits [26,29]. They obtained a vitreous half-life of 2.73 days after a single intravitreal injection of 0.5 mg/0.05 mL in one eye. Another study, in this case with cynomolgus monkeys, was performed with a bilateral single injection of ranibizumab, while testing two different injection doses: 0.5 mg/0.05 mL and 2 mg/0.05 mL [30]. They obtained vitreous half-lives with a one-compartment model of 2.63 and 3.95 days, respectively. The comparison between both groups suggests dose-linear vitreous pharmacokinetics. Aqueous humour, retina, and serum half-lives were also calculated (with a non-compartmental analysis), obtaining values of 2.54, 2.60, and 3.59 days for the injected dose of 0.5 mg, and 2.63, 2.28, and 3.47 days for the 2 mg injected dose, respectively. They also concluded that ranibizumab rapidly distributes to the retina, and it is removed from the vitreous humour through the anterior chamber (ranibizumab was found in the aqueous humour) and the posterior route (ranibizumab was found in both retinal layers). Moreover, they suggested that intraocular metabolism does not play a significant role in the elimination of ranibizumab from the vitreous chamber [30]. Even though Christoforidis and Gaudreault’s studies differ in the monkey species used and the uni/bilateral injection at the dose of 0.5 mg, the estimated vitreous half-lives are comparable (2.73 days vs 2.63 days) [29,30].

#### 2.1.2. Human Studies

A population approach of non-linear pharmacokinetic modelling that is based on serum samples that were collected from patients with AMD enrolled in five clinical trials, receiving from 0.3 to 2 mg per eye of single or multiple intravitreal ranibizumab estimated a vitreous half-life of nine days [22]. Ranibizumab reached maximum serum concentration approximately 0.5 days after intravitreal administration and these concentrations were 90,000 times lower than those that were estimated to be found in the vitreous humour [22]. The estimated serum half-life was two hours, because it is not a full-length antibody and it lacks the FcRn (the neonatal Fc receptor for IgG) that protects from lysosomal degradation, ranibizumab is prone to systemic metabolism [22]. Another study performed three intravitreal injections of ranibizumab (0.5 mg) in AMD patients at monthly intervals. A serum half-life of 5.8 days was calculated after measuring serum levels. They also found that ranibizumab did not demonstrate systemic accumulation between the first and third dose, concluding that ranibizumab is very quickly cleared from the bloodstream [19,31]. 

Aqueous humour half-life was calculated in the study performed by Krohne et al. They included patients that were diagnosed with both clinically significant cataract and macular oedema secondary to AMD, diabetic maculopathy, or retinal vein occlusion. The patients received a single intravitreal injection (0.5 mg) within 40 days before surgery, in which the aqueous samples were obtained, obtaining an aqueous half-life of 7.19 days. Moreover, they performed axial length measurements of the ocular globe in order to correct ranibizumab concentrations for ocular volume, determining that volume differences did not significantly alter the aqueous half-life (7.15 days) [32].

There is no data available with regards to the vitreous levels of ranibizumab after intravitreal injection in humans, and consequently vitreous half-life values are no available. One group applied a mathematical model that was intended for intravitreal pharmacokinetics in rabbits to estimate the vitreous half-life of ranibizumab in humans. They calculated a vitreous half-life for ranibizumab of 4.75 days while using an experimentally determined mean half-life of bevacizumab in humans [33]. 

No significant reductions have been observed in the concentration of systemic VEGF levels between baseline and after intravitreal injections of ranibizumab [34,35,36,37,38] or drug accumulation between doses [31].

### 2.2. Bevacizumab

Bevacizumab is a full monoclonal antibody (with Fc fraction) with an affinity for all subtypes of VEGF-A and systemic indication in the treatment of different types of cancer (breast, colon…) (Table 1). There is no current indication for AMD, but its mechanism of action and its administration at the level of the posterior chamber of the eye at a much lower dose (1.25 mg/0.05 mL) have promoted its off-label use [39]. In addition, the possibility of splitting up the vial in the pharmacy departments reduces the cost of the treatment in comparison with the other two drugs that do have the indication [40].

#### 2.2.1. Animal Studies

Bevacizumab vitreous half-life has been estimated at 4.32 days, following a 1.25 mg/0.05 mL unilateral intravitreal injection in rabbit eyes (non-compartmental analysis), with concentrations that remain above 10 µg/mL for 30 days [41]. The estimated half-life was 4.88 days and 6.8 days in the serum while using the same non-compartment model in the aqueous humour [41]. This data is consistent with the intravitreal pharmacokinetics that were analysed by molecular imaging with I-125-bevacizumab in a rabbit model (*t*_1/2_ = 4.22 days, two-compartment model) [26]. Bakri et al. found that bevacizumab concentration was higher in the aqueous humour of the fellow eye (uninjected) than in the vitreous, concluding that bevacizumab enters the fellow eye from the systemic circulation through the anterior route, reaching the aqueous humour first before diffusing into the vitreous, rather than entering through the choroidal blood flow [41]. However, very low concentrations of bevacizumab were found in the aqueous and vitreous humour of the uninjected eye, so this conclusion must be taken with caution.

Higher vitreous half-life values (5.95 days) were obtained in the study that was performed by Nomoto et al. in a rabbit model (unilateral injection). They also measured the amount of bevacizumab in iris/ciliary body and retina/choroids, obtaining half-lives in these tissues of 5.74 and 6.23 days, respectively. However, the higher half-lives that were obtained can be explained, because they performed an Enzyme-Linked ImmunoSorbent Assay (ELISA) that detected all bevacizumab, i.e., free bevacizumab, VEGF-bevacizumab complex and fragments of bevacizumab molecules, whereas the majority of the studies only measure free bevacizumab levels, as they have been considered as a good representation of total drug concentration. Moreover, their first sample taken was seven days after injection, so early data is missing in this study, which could counterfeit the results [42].

Another study has found even higher half-lives in vitreous, aqueous humour, and serum (6.61 days, 6.51 days, 5.87 days, non-compartmental analysis) when compared to the other studies of 1.25 mg/0.05 mL bevacizumab unilateral injection in rabbit models [43]. They used New Zealand rabbits instead of Dutch-belted rabbits, together with a high sensitivity ELISA (detection limit 0.001 ng/mL) detection kit, which could explain the differences that were found. However, when compared to Bakri’s work, both of the studies found that the maximum concentration was achieved at one day post-injection in the vitreous humour and after eight days in serum [41,43]. One great advantage of this study is that anti-bevacizumab antibodies in serum were also measured, concluding that these anti-bevacizumab antibodies cannot have an important effect on bevacizumab concentration due to their low concentration [43].

The intravitreal half-life estimated in owl monkeys of 1.25 mg/0.05 mL I-124-bevacizumab intravitreal injection was 3.6 days, with detectable concentrations up to 28 days [26]. The pharmacokinetics of bevacizumab in cynomolgus monkeys was also tested by ELISA, but in this case only aqueous humour and serum samples were collected, obtaining half-lives of 2.8 and 12.3 days, respectively [44], preventing the comparison of both studies.

No activity was detected in other parts of the rabbit body apart from the ocular globe in the studies that were performed by Christoforidis et al. with I-125 radiolabelled antibodies bevacizumab, ranibizumab, and aflibercept [26,45]. However, these results are inconsistent with other studies, where bevacizumab was detected in the brain, heart, and kidney after a single intravitreal injection [46].

#### 2.2.2. Human Studies

Vitreous half-life was estimated to be 6.7 days following a two-compartment model. Patients received a single dose of 1.25 mg bevacizumab prior to vitrectomy. A peak concentration of 165 µg/mL was reached on the second day after the intravitreal injection [47]. Another author found that the vitreous half-life ranged between 2.5 and 7.3 days, with a mean of 4.9 days, after the administration of 1.25 mg/0.05 mL while using a one-compartmental model. The vitreous samples were taken during pars plana vitrectomy [34]. These results should be taken with caution, as only eleven [47] and three [34] patients, respectively, were used for the pharmacokinetic analysis. Moreover, the fact that vitrectomy was performed due to collateral complications, such as submacular haemorrhage and choroidal neovascularization [47] or cataract extraction [34] must also be taken into account. The latter study also evaluated the serum half-life of bevacizumab, determining it to be 11.3 days [34].

A serum half-life of 18.7 days [19,31] has been estimated after three-single intravitreal doses of 1.25 mg of bevacizumab. Moreover, its systemic exposure was found to be greater than that og ranibizumab or aflibercept, with a serum concentration of 1.58 nM, which is higher than the estimated inhibitory concentration (IC50) for VEGF factor (IC50 = 0.668 nM) [48]. This data suggests the possibility of adverse effects that are usually associated with the intravenous doses of these drugs, and that could appear in patients with AMD, macular oedema, etc. [49,50,51].

Aqueous half-life was estimated to be 9.82 days in humans by non-compartmental analysis. Patients received a single intravitreal injection of 1.5 mg bevacizumab and within 53 days after the injection, an aqueous humour sample was obtained during cataract surgery. The patients were diagnosed with cataract and recurrent macular oedema secondary to AMD. Bevacizumab concentration peaked on the first day, with a mean concentration of 33.3 µg/mL [52].

The same dose (1.5 mg) was administered in another study and was compared to a higher dose of 3 mg. The maximum concentration in the aqueous humour was obtained at one day post-injection for both doses, with an aqueous half-life of 7.85 and 11.69 days for the 1.5 and 3 mg doses, respectively, calculated by one-compartmental analysis. Double dosing induced a significant higher peak concentration at baseline, although the aqueous bevacizumab concentration was not significantly different after six weeks. Therefore, the administration of a double-dose does not significantly increase the duration of action. This study presents several limitations, as that the enrolled patients suffered from different retinal diseases and the injection volume was double for the dose of 3 mg in relation to 1.5 mg, which could affect the pharmacokinetic parameters [53]. 

Regarding the bevacizumab levels in the aqueous humour of the uninjected eye, the study that was conducted by Meyer et al. found that the concentration of bevacizumab was below the ELISA detection limit, so no significant levels are expected to be found in the fellow aqueous chamber [54]. 

### 2.3. Aflibercept

Aflibercept has a different mechanism than the other two. It is a recombinant fusion protein that consists of portions from the extracellular domains of the human VEGF receptors 1 and 2, which are fused with the Fc portion of the human IgG1. Aflibercept has a great affinity for VEGF A, B, and placental growth factor (P1GF) (Table 1). The data on the pharmacokinetics of aflibercept is scarce and mostly refers to animal models in comparison with the other two anti-VEGF drugs. 

#### 2.3.1. Animal Studies

Christoforidis et al. also performed studies with I-124 radiolabelled aflibercept, obtaining a vitreous half-life of 4.58 days after a single intravitreal injection of 2 mg/0.05 mL in Dutch-belted rabbits [45]. Another study found a vitreous half-life of 3.92 days in New Zeeland white rabbits [55]. The differences could be due to the fact that the aflibercept concentration was quantified by an indirect ELISA and the dose injected was lower, 1.2 mg/0.03 mL. In this study, the aqueous humour and retina/choroid half-lives were also calculated, which obtained values of 2 and 2.425 days, respectively [55]. 

Studies in owl monkeys were also performed with I-124 aflibercept, obtaining a vitreous half-life value of 2.44 days [29], whereas the vitreous half-life value was 2.2 days in cynomolgus macaques where aflibercept concentration was measured by ELISA [56].

#### 2.3.2. Human Studies

Serum half-life of aflibercept has been estimated at 11.4 days after three-monthly intravitreal injections of aflibercept (2.0 mg) in an AMD population. The authors found that aflibercept seemed to exhibit systemic drug accumulation between the first and third dose [19,31].

Some of the authors explored the relation between systemic exposure to intravitreal aflibercept injection and systemic pharmacodynamics (blood pressure). They included patients from four different clinical trials. Aflibercept plasma concentrations quickly decreased over a week to concentrations below the LLOQ (15.6 µg/L) once peak concentrations has been achieved within 1–3 days post-dose. Intravitreal administrations were not associated with common adverse effects of intravenous anti-VEGF [57].

The authors of this article suggest, that owing to the intermediate size of aflibercept (between ranibizumab and bevacizumab), the vitreous half-life of aflibercept could be hypothesised to be nine days since no intravitreal pharmacokinetic studies have been performed in humans with aflibercept [58]. A study conducted in five patients with AMD found an aqueous half-life of approximately nine days based on aqueous samples. They also found very low plasma levels, suggesting a lack of substantial plasma exposure [59].

The same author that calculated a vitreous half-life of ranibizumab in humans with a mathematical model, determining a vitreous half-life of 7.13 days for aflibercept following the same procedure [33].

A compiled list of the different pharmacokinetic parameters analysed in the studies of ranibizumab (Table 2), bevacizumab (Table 3), and aflibercept (Table 4) in different species (rabbit, monkey, and human) has been included at the end of this section. The compiled parameters include half-life (*t*_1/2_), time taken to reach maximum concentration (*T*_max_), maximum concentration (*C*_max_), and area under the concentration-time curve (AUC). *C*_max_ is reached very early on due to the rapid distribution of the antibodies through the vitreous humour, so most of the studies assume that their first data point (normally one day post-injection) corresponds to *T*_max_. Therefore, the utility of defining *T*_max_ is sometimes controversial. However, *T*_max_ is shown in the pharmacokinetic tables in order to provide a time reference corresponding to *C*_max_.

## 3. Pharmacokinetic Considerations

Many factors are involved in the pharmacokinetics of anti-VEGF antibodies, from the physiological conditions of the eye, to the surgical procedures or the analytical methods, which allow for their determination.

### 3.1. Eye Physiological Factors

#### 3.1.1. Distribution-Diffusion in the Vitreous Humour

The distribution of drugs in the vitreous humour is conditioned by its intrinsic characteristics, such as volume and composition, as well as by the properties of the drug (charge, molecular weight, and protein binding capacity). The vitreous humour occupies around 80% of the internal volume of the eye, which is around 4 mL in humans, 1.5 mL in rabbits, and 2 mL in monkeys [65]. It is an avascular structure that is mainly composed of a hydrophilic polymer of hyaluronic acid and collagen, which contributes to its consistency and attracts water, which is its majority component (98%). The central part of the vitreous humour has less of these components than the posterior part, which makes it more fluid-like. In addition, hyaluronic acid has a negative charge, meaning that the restrictive diffusion of positively charged molecules may occur [66]. Bevacizumab and ranibizumab are both negatively charged molecules under physiological conditions, therefore their movement should not be restricted in the vitreous humour [24]. However, aflibercept is considered to have a mild positive charge, which might affect its pharmacokinetic properties [25].

On other hand, molecular weight affects drug diffusion, which therefore affects the half-life. The anti-VEGF molecules are rather heavy molecules (149 KDa, 115 KDa, 48 KDa for bevacizumab, aflibercept, and ranibizumab, respectively), so they are expected to have a low intravitreal clearance when compared with small molecules that do not have steric hindrance (in general, intravitreal half-life increases as molecular weight rises above 10,000 Da) [67]. A comparison of the properties of the three anti-VEGF inhibitors can be found in Table 1 [23,24,25].

Moreover, the rheological properties of the vitreous humour change with age, in a process called liquefaction, in which the vitreous humour is turned into a more liquefied state. Liquefaction might increase drug diffusion, especially in those with high molecular weight [67]. When compared to plasma, the concentration and number of proteins in the vitreous humour is low (0.5–1.5 mg/mL in vitreous vs 60–80 mg/mL in plasma) [68]. The main proteins are collagen (a structural protein), albumin, and immunoglobulins (non-structural proteins) [69]. The binding of drugs to proteins might reduce its distribution through the vitreous humour, although this factor does not seem to affect the diffusion of anti-VEGF drugs [70].

#### 3.1.2. Elimination of Drugs from the Vitreous Humour

The elimination of drugs from the vitreous humour can occur via two different routes, either by metabolism or by disposal into the systemic circulation. In the case of the anti-VEGF drugs, they do not appear to suffer metabolism nor degradation in the eye [58,70]. 

After intravitreal injection, the drugs can be removed to the systemic circulation by two routes: the anterior route or the posterior route. The anterior route consists of drug diffusion through the vitreous humour until it reaches the aqueous humour and it is then eliminated through its flow. All the drugs can be eliminated in this way. Various reports have considered that anti-VEGF drugs are mainly eliminated through the anterior route [21,28,32,52,71].

The posterior route consists of the secretion of the drug by the epithelium of the ciliary body, iris, or retinal pigment epithelium [70]. Peters and Heidushka tried to demonstrate that bevacizumab was also eliminated through the posterior route crossing the blood-retinal barrier. They observed that bevacizumab immunoreactivity after the intravitreal injection extended over time to the inner layers of the retina. However, they did not attempt to determine whether or not active transport is involved in this process [72,73]. The effect of active transport through the retina is not yet clear, so the impact that this may have on the drug pharmacokinetics is yet to be defined [74].

### 3.2. Surgical Ocular Procedures (Lensectomy and Vitrectomy)

The distribution and elimination of anti-VEGF drugs from the vitreous are intimately related to several ophthalmic surgical procedures. Laude et al. suggest that cataract operated patients could have a faster clearance of vitreous drugs [67]. However, Krohne et al. found that ocular volume and lens status have no relevant impact on ocular pharmacokinetics and the duration of action of anti-VEGF drugs after comparing VEGF suppression times in phakic (natural lens) and pseudophakic (replaced crystalline lens) human eyes [75]. 

On the other hand, many patients with macular disease who are being treated with anti-VEGF drugs require surgical intervention for complications, such as bleeding in the vitreous. It is known that replacing the gel-like vitreous humour with a less viscous saline or aqueous humour facilitates the transportation of oxygen, as well as the clearance of VEGF inhibitors and cytokines, reducing oedema and retinal neovascularisation [76,77]. Additionally, the surgical procedure itself and the use of silicone oil as vitreous replacement can influence drug pharmacokinetics [78]. 

There are few studies that correlate the effect of vitrectomy with the pharmacokinetics and these give very different results. A study performed on rabbits with labelled mAbs with I-124 demonstrated a reduction in the half-life of anti-VEGF drugs after vitrectomy and lensectomy, going from 4.22 to 2.30 and 2.08 days, respectively, for bevacizumab and from 2.81 to 2.13 and 1.79 days, respectively, for ranibizumab [60]. The same author also quantified the serum concentration of bevacizumab in rabbits, finding that the serum levels initially increased following the vitrectomy, but determining that there were not any significant differences later on [61]. On the contrary, other authors did not find significant differences on the vitreous half-life on injected bevacizumab in non-vitrectomised vs vitrectomised eyes in rabbit eyes (7.06 days vs 6.99 days) [62], which suggested that VEGF is a complex molecule that is not restricted to the elimination by diffusion. However, they did find that vitrectomy affected the PK parameters in the initial distribution phase in a two-phase fitting [62]. Another study that was published by the same author on ranibizumab also showed no differences between the vitreous half-life in normal rabbit eyes (2.75 days) and following the vitrectomy (2.51 days) [27]. In this case, the parameters were established according to one-phase kinetics. 

After a vitrectomy, filling the vitreous cavity with a tamponade, such as silicone oil, is a common procedure. The impact of silicone-oil filled eyes in the pharmacokinetics of injected bevacizumab was studied, observing longer *T*_max_, smaller *C*_max_, and relatively sustained bevacizumab levels in the ocular tissues in comparison with non-vitrectomised rabbit eyes [79].

Niwa et al. calculated the aqueous half-life of intravitreally injected ranibizumab and aflibercept in macaque eyes, even though the majority of the studies were performed in rabbit model. They found that the aqueous half-life was reduced after the vitrectomy (from 2.3 to 1.4 days for ranibizumab and from 2.2 to 1.5 days for aflibercept) [56]. However, these results must be taken with caution, due to the fact that the aqueous half-life might be not comparable to the vitreous half-life. 

In summary, there is evidence of a decrease in the half-life of intravitreal injected antibodies after vitrectomy is performed, although it is not quite clear whether or not these differences are relevant enough to change the injection interval of anti-VEGF antibodies [64,76]. Moreover, this decrease is higher when the vitrectomy is performed in combination with a lensectomy. However, these results come from animal studies and their translation to humans is still controversial, which is mainly due to the anatomic and physiological differences between the species [76]. 

### 3.3. Analytical Methods Used in Pharmacokinetic Studies

The assessment of pharmacokinetic parameters for a drug administered by intravitreal route poses a challenge. It is not easy to obtain periodic samples of vitreous or aqueous fluids due to the invasive nature of the method. Moreover, when trying to assess drug systemic levels, the exposure may be low, or the technique does not offer the sensitivity that is required to enable pharmacokinetic evaluations of antibodies. Most of the reported assays are based on ELISA (Enzyme-Linked ImmunoSorbent Assay) assays, which are considered the “gold standard” method used for the measurement of monoclonal antibodies [22,30,34]. Although there are a large variety of ELISA methods available for anti-VEGF antibodies determination, most of the work in this field relies on an indirect determination by VEGF, where factors, such as the type of VEGF, or the binding affinity, might have a big influence. Out of the three, ranibizumab is the one that requires a higher sensitivity and a more specific detection method, since the ranibizumab serum levels are often lower than the levels that can be detected by conventional methods [21,30]. The pharmacokinetic profile of Fab antibodies (such as ranibizumab) is characterised by a long elimination of the vitreous half-life and a rapid elimination from the systemic circulation [80]. Molecules containing a Fc region, such as bevacizumab or aflibercept, have greater systemic half-lives [58], because they are protected from proteolytic catabolism by binding to the neonatal Fc receptor (FcRn). However, the impact of FcRn receptor on the intravitreal pharmacokinetics is still unclear [70]. Additionally, high sensitivity ELISA methods require for drug samples to be diluted within the detection range, which can add some inaccuracy.

Lowe et al. developed a novel electrochemiluminescence assay (ECLA) that allowed for a more sensitive determination of ranibizumab in serum. This assay was first used to support some clinical trials, offering 67 times more sensitivity than a conventional ELISA (20 ng/mL) [81] and with a reporting range of 0.3–24 ng/mL [82]. More recently, the same authors have presented another novel method that utilises a high-affinity monoclonal anti-ranibizumab-VEGF-complexes antibody (MARA) to measure ranibizumab in human serum. The assay format uses a semi-homogeneous solution that specifically binds to the ranibizumab-VEGF complex, but neither one alone. This new ELISA method has a lower limit of quantification of 15 pg/mL in human serum [83].

There are still a few studies have attempted to improve the detection method, even though most of the studies quantify the anti-VEGF drugs concentration by immunoassays. Dickmann et al. assessed the ability of fluorophotometry to measure the intravitreal pharmacokinetics of fluorescently-labelled ranibizumab in the rabbit and compared the results to those that were obtained using ELISA in previous publications, obtaining similar results [84].

Christoforidis et al. tried a different approach by labelling bevacizumab, ranibizumab, and aflibercept with a radionuclide, such as I-124, to evaluate the pharmacokinetics of the intravitreally injected anti-VEGF drugs by PET/CT [26,45,60]. The great advantage of this method in comparison to the traditional ones using ELISA is that the vitreous anti-VEGF antibodies levels can be controlled without needing to sacrifice the animals at determined time intervals or without taking invasive samples of the vitreous humour. 

HPLC (High Performance Liquid Chromatography) is a fast and low-cost quantification method, however it is not commonly used in antibodies determinations in biological samples. Giannos et al. tried to correlate ELISA analytical methods to SE-HPLC (size exclusion high performance liquid chromatography) on in vitro studies, showing a close and significant correlation between them. Their SE-HLPC method uses a new marketed column designed for antibodies with a lower limit of detection (LOD) of 2.19 ng/mL and a lower limit of quantification (LLQ) of 8.79 ng/mL for bevacizumab and ranibizumab. Aflibercept LOD and LLQ were 8.79 and 17.578 ng/mL, respectively [85]. No in vivo studies were found that used HPLC as an analytical method for anti-VEGF drugs.

## 4. Outlooks

**Figure 1:**
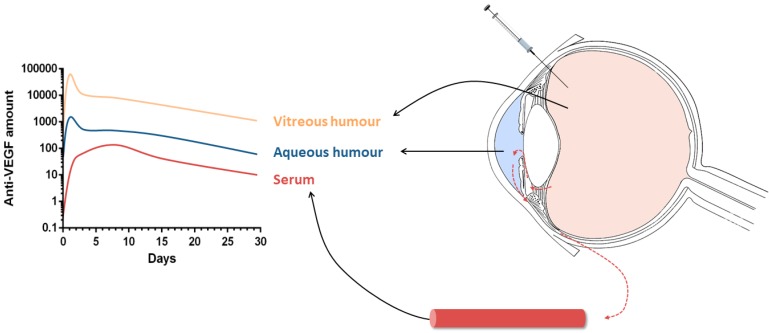
Scheme of the pharmacokinetic profiles after anti-VEGF antibodies intravitreal injection.

All of the pharmacokinetic studies centre their reports on the half-lives of the anti-VEGF drugs in different compartments (vitreous, aqueous humour, or serum) (Figure 1). Their objective is to explain the route of elimination of the drug from the eye, in the case of animal studies, or to relate the findings to possible adverse drug effects when entering the systemic circulation. 

Only one study has compared the pharmacokinetics of the three anti-VEGF antibodies that were used in clinical practice [19]. Avery et al. compared the systemic exposure and the suppression of VEGF in plasma. Ranibizumab showed the least systemic exposure, whereas bevacizumab presented the highest with a 35-fold increase in AUC as compared to ranibizumab. These differences further increase after the third dose. Aflibercept appears to have the greatest suppression of free plasma VEGF out of the three, with serum concentrations that exceed its IC50 value (0.068 nM) at three hours post-injection and remain above this for seven days. In contrast, ranibizumab mean trough levels remained similar to the baseline [19].

However, no study has extensively examined the ocular pharmacokinetics of anti-VEGF antibodies in humans and their relation to the frequency of intravitreal doses. The establishment of the actual dosage regimens is mainly based on the activity of the disease that is assessed by OCT imaging or visual acuity and not on the pharmacokinetics of the drugs. An in vitro model in aqueous humour tried to associate the VEGF-A suppression times with the administration times, which suggested that individual dosing strategies are possible with a range of suppression of 26 to 69 days [86,87]. In humans, only one study determined levels of unbound aflibercept in a case series with seven patients that were treated over a six-month period with aflibercept and unbound VEGF-A in aqueous humour remained stable after every month and second month of intravitreal injections, supporting that bimonthly administrations may be enough in those patients that were treated with aflibercept [88].

Population pharmacokinetic analysis allows for the drug time-course profiles and the response dynamics over time to be characterised in a more precise manner. It also allows for the identification of the intrinsic and extrinsic factors that might be related to the observed drug exposure or response [86]. Population analysis, which is also known as non-linear mixed effects modelling, considers the structural pharmacokinetic or pharmacokinetic/pharmacodynamic models and stochastic models in order to account for inter-individual and/or inter-occasion variability and residual unexplained error [89,90,91,92]. Regulatory authorities have actually acknowledged the relevance of this discipline for drug approval and its value for an optimal dose selection in the special subgroups of the population [93,94]. However, these models are not used in the development of new agents for AMD, and only one author has applied the concepts of non-linear mixed effects modelling for the characterisation of the pharmacokinetic time-course profile of ranibizumab in this disease [22].

## 5. Conclusions

At present, the available pharmacokinetic data on anti-VEGF drugs after intravitreal administration are still limited, despite the fact that these molecules are the standard treatment for AMD. In recent years, many studies have been carried out in order to determine the main pharmacokinetic parameters of anti-VEGF antibodies in different animal species and humans, although the differences in the methods of determination, in the samples analysed, in the time points taken, and the compartmental analysis, etc., make it difficult to attain standardised values for each anti-VEGF antibody. We believe that this comprehensive review will be of great use to research groups working on the pharmacokinetics of intravitreally administered VEGF inhibitors, although further studies are necessary in order to improve the knowledge in this area.

## Figures and Tables

**Table 1:** Properties of anti-vascular endothelial growth factor (VEGF) antibodies for Age-Related Macular Degeneration (AMD). Data from [23,24,25].

Properties	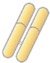	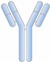	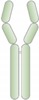
	Ranibizumab	Bevacizumab	Aflibercept
Class	Antibody fragment	Monoclonal antibody	Fusion protein
MW (KDa)	48	149	115
Net charge	Negative	Negative	Slightly positive
Binding target	VEGF-A	VEGF-A	VEGF-A, VEGF-B, PIGF
*K*_D_ for VEGF_165_ (pM)	46	58	0.49

*M*_w_ = molecular weight; *K*_D_ = equilibrium dissociation constant.

**Table 2:** Pharmacokinetic parameters of intravitreal ranibizumab in different species.

Model	Injected Dose	Determination	Sensitivity	PK Model	Sample	Time Points	Normal Eyes				Vitrectomy			Aphakia	Observations	Ref.
							*t*_1/2_	*T*_max_	*C*_max_	AUC	*t*_1/2_	*C*_max_	AUC	*t*_1/2_		
Dutch-belted rabbits	0.5 mg/0.05 mL	CLIA	LLOQ = 0.375 ng/mL	NC	VH	1, 3, 8, 15, 29 days	2.88 days	1 day	162 µg/mL						No detection in serum	[21]
					AH		2.84 days	3 days	17.9 µg/mL							
					Serum											
Dutch-belted rabbits	0.5 mg/0.05 mL	PET (I-124)		2C	VH	0, 2, 5, 7, 14, 21, 28, 35 days	2.81 days	0 h							No detection in other organs	[26]
					AH											
					Serum											
Dutch-belted rabbits	0.5 mg/0.05 mL	PET (I-124)		2C	VH	0, 2, 5, 7, 14, 21, 28, 35 days	2.81 days				2.13 days			1.79 days		[60]
					AH											
					Serum											
New Zealand rabbits	0.625 mg/0.05 mL	ELISA	0.78 ng/mL	1C	VH	1, 8 h; 1, 2, 4, 7, 14, 21, 30, 42, 50, 60 days	2.9 days	1 h	1280 µg/mL						Bilateral injection	[28]
			0.78 ng/mL	NC	AH		3 days	48 h	57.1 µg/mL							
			7.8 ng/mL	NC	Serum			24 h	0.055 µg/mL							
New Zealand rabbits	0.25 mg/0.025 mL	ELISA	LLOQ = 0.375 ng/mL	1C	VH	1 h or 1, 2, 5, 14, 30 days	2.75 days	1 h	91.61 µg/mL		2.51 days	118.01 µg/mL				[27]
					AH			1 h	20.38 µg/mL			21.7 µg/mL				
					Serum											
Owl monkeys	0.5 mg/0.05 mL	PET (I-124)		2C	VH	0, 1, 2, 4, 8, 14, 21, 28, 35 days	2.73 days									[29]
					AH											
					Serum	1, 2, 4, 8, 12 h; 1, 2, 4, 8, 14, 21, 28, 35 days		24 h	0.47 ng/mL							
Cynomolgus macaques	0.5 mg/0.05 mL	ELISA	1.5 ng/mL	1C	VH	6 h, 2, 3, 5, 8, 11 days	2.63 days	6 h	169 µg/mL						Bilateral injection	[30]
			1.5 ng/mL	NC	AH		2.54 days	6 h	116 µg/mL							
			15.6 ng/mL	NC	Serum	2, 6, 12, 24, 36, 48 h; 4–11 days	3.59 days	6 h	150 µg/mL							
Cynomolgus macaques	2 mg/0.05 mL	ELISA	1.5 ng/mL	1C	VH	6 h, 2, 3, 5, 8, 11 days	3.95 days	1 day	612 µg/mL						Bilateral injection	[30]
			1.5 ng/mL	NC	AH		2.63 days	1 day	478 µg/mL							
			15.6 ng/mL	NC	Serum	2, 6, 12, 24, 36, 48 h; 4–11 days	3.47 days	6 h	616 µg/mL							
Cynomolgus macaques	0.25 mg/0.05 mL	ELISA	LLOD = 156 pg/mL	1C	VH	1, 3 days; 1–8 weeks										[56]
					AH		2.3 days	1 day	51.3 µg/mL	171 days ·µg/mL	1.4 days	41.8 µg/mL	154 days ·µg/mL			
					Serum											
Human	0.5 mg/0.05 mL	ELISA	10–1000 ng/mL	1C	VH	1–37 days										[32]
					AH		7.19 days	1 day	56.1 µg/mL							
					Serum											
Human	Variable	CLIA	LLOQ = 0.3 ng/mL	1C	VH	Variable	9 days									[22]
					AH											
					Serum		2 h									
Human	0.5 mg/0.05 mL	ELISA	LLOQ = 15 pg/mL	NC	VH	3 h; 1, 3, 7, 28 days										[19,31]
					AH											
					Serum		5.8 days		0.11 nM	0.46 h·nM						

CLIA = Chemoluminiscent immunoassay; ELISA = Enzyme-Linked ImmunoSorbent Assay; PET = Positron Emission Tomography; LLOD = Lower Limit of Detection, LLOQ = Lower Limit of Quantification, 1C = One-compartment model; 2C = Two-compartment model; NC = Non-compartment model; VH = vitreous humour; AH = aqueous humour; *t*_1/2_ = half-life; *T*_max_ = time taken to reach maximum concentration; *C*_max_ = maximum concentration; AUC = area under the curve.

**Table 3:** Pharmacokinetic parameters of intravitreal bevacizumab in different species.

Model	Injected Dose	Determination	Sensitivity	PK Model	Sample	Time Points	Normal Eyes				Vitrectomy			Aphakia	Observations	Ref.
							*t*_1/2_	*T*_max_	*C*_max_	AUC	*t*_1/2_	*C*_max_	AUC	*t*_1/2_		
Dutch-belted rabbits	1.25 mg/0.05 mL	CLIA	LLOQ = 0.0625 ng/mL	NC	VH	1, 3, 8, 15, 29 days	4.32 days	1 day	400 µg/mL							[41]
					AH		4.88 days	3 days	37.7 µg/mL							
					Serum		6.86 days	8 days	3.33 µg/mL							
Dutch-belted rabbits	1.25 mg/0.05 mL	ELISA	LLOQ = 0.1 ng/mL		VH	1, 2, 4, 12 weeks	5.95 days	7 days	59.7308 µg/mL							[42]
					AH			7 days	373.6 ng/mL							
					Serum		12.95 days	14 days	2.0872 µg/mL							
Dutch-belted rabbits	1.25 mg/0.05 mL	PET (I-124)		2C	VH	0, 2, 5, 7, 14, 21, 28, 35 days	4.22 days	0 h							No detection in other organs	[26]
					AH											
					Serum											
Dutch-belted rabbits	1.25 mg/0.05 mL	PET (I-124)		2C	VH	0, 2, 5, 7, 14, 21, 28, 35 days	4.22 days				2.30 days			2.08 days		[60]
					AH											
					Serum											
Dutch-belted rabbits	1.25 mg/0.05 mL	ELISA	LLOD = 10 ng/mL	1C	VH	2, 4, 7, 10, 14, 21, 28, 35 days										[61]
					AH											
					Serum		6.69 days	6.4 days	6.22 µg/mL	69.2 d·µg/mL	2.80 days	6.19 µg/mL	84.1 d·µg/mL	1.41 days		
New Zealand rabbits	1.25 mg/0.05 mL	ELISA	LLOD = 0.01 ng/mL	NC	VH	1, 3, 8, 15, 29 days	6.61 days	1 day	406.25 µg/mL							[43]
					AH		6.51 days	1 day	5.835 µg/mL							
					Serum		5.87 days	8 days	0.413 µg/mL							
New Zealand rabbits	1.25 mg/0.05mL	ELISA	LLOQ = 0.0625 ng/mL	1C	VH	1 h; 1, 2, 5, 14, 30 days	7.06 days	1 h	1021.54 mg/mL		6.99 days					[62]
					AH			2 days	121 mg/mL							
					Serum											
New Zealand rabbits	0.025 mL	PET (Zr-89)			VH	5–60 min; 4, 24, 48, 120, 144 h	3.51 days									[63]
					AH											
					Serum											
Owl monkeys	1.25 mg/0.05 mL	PET (I-124)		2C	VH	0, 1, 2, 4, 8, 14, 21, 28, 35 days	3.60 days									[29]
					AH											
					Serum	1, 2, 4, 8, 12 h; 1, 2, 4, 8, 14, 21, 28, 35 days		3.5 days	7.80 ng/mL							
Cynomolgus macaques	1.25 mg/0.05 mL	ELISA	LLOD = 0.156 ng/mL		VH	1, 3, 7 d; 2, 4, 6, 8 weeks										[44]
					AH		2.8 days	1 day	49.500 µg/mL							
					Serum		12.3 days	7 days	1.430 µg/mL							
Cynomolgus macaques	1.25 mg/0.05 mL	ELISA	7.8–1000 pg/mL		VH	1, 3, 7 d; 2, 4, 6, 8 weeks										[64]
					AH			1 day	10.8 μg/mL		1.5 days					
					Serum		5.9 days	1 day	42.2 ng/mL							
Human	1.25 mg/0.05 mL	ELISA	LLOQ = 313 pg/mL	NC	VH	3 h; 1, 3, 7, 28 days										[19,31]
					AH											
					Serum		18.7 days		0.76 nM	16.10 h·nM						
Human	1.25 mg/0.05 mL	ELISA		2C	VH	1–101 days	6.7 days	2 days	165 µg/mL	2036 d·µg/mL						[47]
					AH											
					Serum											
Human	1.25 mg/0.05 mL	ELISA	6.25 ng/mL		VH	Variable	4.9 days				0.66 day					[34]
					AH											
					Serum		11.3 days									
Human	1.5 mg	ELISA		1C	VH	1–53 days										[52]
					AH		9.82 days	1 day	33.3 µg/mL							
					Serum											
Human	1.5 mg	ELISA		1C	VH	1–60 days										[53]
					AH		7.85 days	1 day	14.86 µg/mL							
					Serum											
Human	3 mg	ELISA		1C	VH	1–60 days										[53]
					AH		11.69 days	1 day	27.74 µg/mL							
					Serum											

CLIA = Chemoluminiscent immunoassay; ELISA = Enzyme-Linked ImmunoSorbent Assay; PET = Positron Emission Tomography; LLOD = Lower Limit of Detection, LLOQ = Lower Limit of Quantification, 1C = One-compartment model; 2C = Two-compartment model; NC = Non-compartment model; VH = vitreous humour; AH = aqueous humour; *t*_1/2_ = half-life; *T*_max_ = time taken to reach maximum concentration; *C*_max_ = maximum concentration; AUC = area under the curve.

**Table 4:** Pharmacokinetic parameters of intravitreal aflibercept in different species.

Model	Injected dose	Determination	Sensitivity	PK Model	Sample	Time Points	Normal Eyes				Vitrectomy			Aphakia	Observations	Ref.
							*t*_1/2_	*T*_max_	*C*_max_	AUC	*t*_1/2_	*C*_max_	AUC	*t*_1/2_		
Dutch-belted rabbits	2 mg/0.05 mL	PET (I-124)		1C	VH	0, 2, 5, 7, 14, 21, 28, 35 days	4.58 days	0 h							No detection in other organs	[45]
					AH											
					Serum											
Owl monkeys	2 mg/0.05 mL	PET (I-124)		2C	VH	0, 1, 2, 4, 8, 14, 21, 28, 35 days	2.44 days									[29]
					AH											
					Serum	1, 2, 4, 8, 12 h; 1, 2, 4, 8, 14, 21, 28, 35 days		2 days	3.50 ng/mL							
Cynomolgus macaques	2 mg/0.05 mL	ELISA	LLOD = 156 pg/mL	1C	VH	1, 3 days; 1–8 weeks										[56]
					AH		2.2 days	1 day	74 µg/mL	174 d·µg/mL	1.5 days	68 µg/mL	124 d·µg/mL			
					Serum											
Human	2 mg/0.05 mL	ELISA	LLOQ = 1000 pg/mL	NC	VH	3 h; 1, 3, 7, 28 days										[19,31]
					AH											
					Serum		11.4 days		0.45 nM	4.32 h·nM						
Human	2 mg	ELISA			VH	4 h; 1, 3, 7, 14, 28 days										[59]
					AH		11 days	4 h	64.4 mg/L							
					Serum			4 h	0 mg/L							

CLIA = Chemoluminiscent immunoassay; ELISA = Enzyme-Linked ImmunoSorbent Assay; PET = Positron Emission Tomography; LLOD = Lower Limit of Detection, LLOQ = Lower Limit of Quantification, 1C = One-compartment model; 2C = Two-compartment model; NC = Non-compartment model; VH = vitreous humour; AH = aqueous humour; *t*_1/2_ = half-life; *T*_max_ = time taken to reach maximum concentration; *C*_max_ = maximum concentration; AUC = area under the curve.

## References

[1] Pascolini D., Mariotti S.P. (2012). Global estimates of visual impairment: 2010. Br. J. Ophthalmol..

[2] Wong W.L., Su X., Li X., Cheung C.M.G., Klein R., Cheng C.-Y., Wong T.Y. (2014). Global prevalence of age-related macular degeneration and disease burden projection for 2020 and 2040: A systematic review and meta-analysis. Lancet Glob. Health.

[3] Ferris F.L., Wilkinson C.P., Bird A., Chakravarthy U., Chew E., Csaky K., Sadda S.R. (2013). Beckman Initiative for Macular Research Classification Committee Clinical classification of age-related macular degeneration. Ophthalmology.

[4] De Jong P.T.V.M. (2006). Age-related macular degeneration. N. Engl. J. Med..

[5] Rohrer B., Frazer-Abel A., Leonard A., Ratnapriya R., Ward T., Pietraszkiewicz A., O’Quinn E., Adams K., Swaroop A., Wolf B.J. (2019). Association of age-related macular degeneration with complement activation products, smoking, and single nucleotide polymorphisms in South Carolinians of European and African descent. Mol. Vis..

[6] Empeslidis T., Storey M., Giannopoulos T., Konidaris V., Tranos P.G., Panagiotou E.S., Voudouragkaki I.C., Konstas A.G. (2019). How Successful is Switching from Bevacizumab or Ranibizumab to Aflibercept in Age-Related Macular Degeneration? A Systematic Overview. Adv. Ther..

[7] Pershing S., Talwar N., Armenti S.T., Grubbs J., Rosenthal J.M., Dedania V.S., Stein J.D. (2019). Use of Bevacizumab and Ranibizumab for Wet Age-Related Macular Degeneration: Influence of CATT Results and Introduction of Aflibercept. Am. J. Ophthalmol..

[8] Cioffi C.L., Johnson G., Petrukhin K., Desai M.C. (2016). Recent Developments in Agents for the Treatment of Age-related Macular Degeneration and Stargardt Disease. 2016 Medicinal Chemistry Reviews.

[9] Neumann R., Barequet D. (2019). The gap between the need for novel retinal drug delivery methods, technologies in R&D phase, and approved ocular drug delivery technologies. Drug Discov. Today.

[10] Brown D.M., Michels M., Kaiser P.K., Heier J.S., Sy J.P., Ianchulev T. (2009). ANCHOR Study Group Ranibizumab versus verteporfin photodynamic therapy for neovascular age-related macular degeneration: Two-year results of the ANCHOR study. Ophthalmology.

[11] Rosenfeld P.J., Brown D.M., Heier J.S., Boyer D.S., Kaiser P.K., Chung C.Y., Kim R.Y. (2006). MARINA Study Group Ranibizumab for neovascular age-related macular degeneration. N. Engl. J. Med..

[12] Heier J.S., Brown D.M., Chong V., Korobelnik J.-F., Kaiser P.K., Nguyen Q.D., Kirchhof B., Ho A., Ogura Y., Yancopoulos G.D. (2012). Intravitreal aflibercept (VEGF trap-eye) in wet age-related macular degeneration. Ophthalmology.

[13] Schmidt-Erfurth U., Garcia-Arumi J., Bandello F., Berg K., Chakravarthy U., Gerendas B.S., Jonas J., Larsen M., Tadayoni R., Loewenstein A. (2017). Guidelines for the Management of Diabetic Macular Edema by the European Society of Retina Specialists (EURETINA). OPH.

[14] García-Layana A., Figueroa M.S., Arias L., Araiz J., Ruiz-Moreno J.M., García-Arumí J., Gómez-Ulla F., López-Gálvez M.I., Cabrera-López F., García-Campos J.M. (2015). Individualized Therapy with Ranibizumab in Wet Age-Related Macular Degeneration. J. Ophthalmol..

[15] (2015). Cost comparison table of anti-VEGF therapies for W-AMD. Aflibercept (Eylea): Treatment of Neovascular (Wet) Age-Related Macular Degeneration (wAMD).

[16] Okada M., Kandasamy R., Chong E.W., McGuiness M., Guymer R.H. (2018). The Treat-and-Extend Injection Regimen Versus Alternate Dosing Strategies in Age-related Macular Degeneration: A Systematic Review and Meta-analysis. Am. J. Ophthalmol..

[17] Haga A., Kawaji T., Ideta R., Inomata Y., Tanihara H. (2018). Treat-and-extend versus every-other-month regimens with aflibercept in age-related macular degeneration. Acta Ophthalmol..

[18] Al-Zamil W.M., Yassin S.A. (2017). Recent developments in age-related macular degeneration: A review. Clin. Interv. Aging.

[19] Avery R.L., Castellarin A.A., Steinle N.C., Dhoot D.S., Pieramici D.J., See R., Couvillion S., Nasir M.A., Rabena M.D., Le K. (2014). Systemic pharmacokinetics following intravitreal injections of ranibizumab, bevacizumab or aflibercept in patients with neovascular AMD. Br. J. Ophthalmol..

[20] Bhagat R., Zhang J., Farooq S., Li X.-Y. (2014). Comparison of the release profile and pharmacokinetics of intact and fragmented dexamethasone intravitreal implants in rabbit eyes. J. Ocul. Pharmacol. Ther..

[21] Bakri S.J., Snyder M.R., Reid J.M., Pulido J.S., Ezzat M.K., Singh R.J. (2007). Pharmacokinetics of intravitreal ranibizumab (Lucentis). Ophthalmology.

[22] Xu L., Lu T., Tuomi L., Jumbe N., Lu J., Eppler S., Kuebler P., Damico-Beyer L.A., Joshi A. (2013). Pharmacokinetics of ranibizumab in patients with neovascular age-related macular degeneration: A population approach. Investig. Ophthalmol. Vis. Sci..

[23] Papadopoulos N., Martin J., Ruan Q., Rafique A., Rosconi M.P., Shi E., Pyles E.A., Yancopoulos G.D., Stahl N., Wiegand S.J. (2012). Binding and neutralization of vascular endothelial growth factor (VEGF) and related ligands by VEGF Trap, ranibizumab and bevacizumab. Angiogenesis.

[24] Li S.K., Liddell M.R., Wen H. (2011). Effective electrophoretic mobilities and charges of anti-VEGF proteins determined by capillary zone electrophoresis. J. Pharm. Biomed. Anal..

[25] Holash J., Davis S., Papadopoulos N., Croll S.D., Ho L., Russell M., Boland P., Leidich R., Hylton D., Burova E. (2002). VEGF-Trap: A VEGF blocker with potent antitumor effects. Proc. Natl. Acad. Sci. USA.

[26] Christoforidis J.B., Carlton M.M., Knopp M.V., Hinkle G.H. (2011). PET/CT imaging of I-124-radiolabeled bevacizumab and ranibizumab after intravitreal injection in a rabbit model. Investig. Ophthalmol. Vis. Sci..

[27] Ahn S.J., Ahn J., Park S., Kim H., Hwang D.J., Park J.H., Park J.Y., Chung J.Y., Park K.H., Woo S.J. (2014). Intraocular pharmacokinetics of ranibizumab in vitrectomized versus nonvitrectomized eyes. Investig. Ophthalmol. Vis. Sci..

[28] Gaudreault J., Fei D., Beyer J.C., Ryan A., Rangell L., Shiu V., Damico L.A. (2007). Pharmacokinetics and retinal distribution of ranibizumab, a humanized antibody fragment directed against VEGF-A, following intravitreal administration in rabbits. Retina.

[29] Christoforidis J.B., Briley K., Binzel K., Bhatia P., Wei L., Kumar K., Knopp M.V. (2017). Systemic Biodistribution and Intravitreal Pharmacokinetic Properties of Bevacizumab, Ranibizumab, and Aflibercept in a Nonhuman Primate Model. Investig. Ophthalmol. Vis. Sci..

[30] Gaudreault J., Fei D., Rusit J., Suboc P., Shiu V. (2005). Preclinical pharmacokinetics of Ranibizumab (rhuFabV2) after a single intravitreal administration. Investig. Ophthalmol. Vis. Sci..

[31] Avery R.L., Castellarin A.A., Steinle N.C., Dhoot D.S., Pieramici D.J., See R., Couvillion S., Nasir M.A., Rabena M.D., Maia M. (2017). Systemic pharmacokinetics and pharmacodynamics of intravitreal aflibercept, bevacizumab, and ranibizumab. Retina.

[32] Krohne T.U., Liu Z., Holz F.G., Meyer C.H. (2012). Intraocular pharmacokinetics of ranibizumab following a single intravitreal injection in humans. Am. J. Ophthalmol..

[33] Stewart M.W. (2011). What are the half-lives of ranibizumab and aflibercept (VEGF Trap-eye) in human eyes? Calculations with a mathematical model. Eye Rep..

[34] Moisseiev E., Waisbourd M., Ben-Artsi E., Levinger E., Barak A., Daniels T., Csaky K., Loewenstein A., Barequet I.S. (2014). Pharmacokinetics of bevacizumab after topical and intravitreal administration in human eyes. Graefes Arch. Clin. Exp. Ophthalmol..

[35] Tew W.P., Gordon M., Murren J., Dupont J., Pezzulli S., Aghajanian C., Sabbatini P., Mendelson D., Schwartz L., Gettinger S. (2010). Phase 1 study of aflibercept administered subcutaneously to patients with advanced solid tumors. Clin. Cancer Res..

[36] Wang X., Sawada T., Sawada O., Saishin Y., Liu P., Ohji M. (2014). Serum and plasma vascular endothelial growth factor concentrations before and after intravitreal injection of aflibercept or ranibizumab for age-related macular degeneration. Am. J. Ophthalmol..

[37] Zehetner C., Kirchmair R., Huber S., Kralinger M.T., Kieselbach G.F. (2013). Plasma levels of vascular endothelial growth factor before and after intravitreal injection of bevacizumab, ranibizumab and pegaptanib in patients with age-related macular degeneration, and in patients with diabetic macular oedema. Br. J. Ophthalmol.

[38] Zehetner C., Kralinger M.T., Modi Y.S., Waltl I., Ulmer H., Kirchmair R., Bechrakis N.E., Kieselbach G.F. (2015). Systemic levels of vascular endothelial growth factor before and after intravitreal injection of aflibercept or ranibizumab in patients with age-related macular degeneration: A randomised, prospective trial. Acta Ophthalmol..

[39] Reimbursement by a National Healthcare Insurance System of a Medicinal Product for a Use Not Covered by Its Marketing Authorisation (Off-Label Use). http://curia.europa.eu/juris/document/document.jsf?text=&docid=207947&pageIndex=0&doclang=en&mode=req&dir=&occ=first&part=1.

[40] Dakin H.A., Wordsworth S., Rogers C.A., Abangma G., Raftery J., Harding S.P., Lotery A.J., Downes S.M., Chakravarthy U., Reeves B.C. (2014). Cost-effectiveness of ranibizumab and bevacizumab for age-related macular degeneration: 2-year findings from the IVAN randomised trial. BMJ Open.

[41] Bakri S.J., Snyder M.R., Reid J.M., Pulido J.S., Singh R.J. (2007). Pharmacokinetics of intravitreal bevacizumab (Avastin). Ophthalmology.

[42] Nomoto H., Shiraga F., Kuno N., Kimura E., Fujii S., Shinomiya K., Nugent A.K., Hirooka K., Baba T. (2009). Pharmacokinetics of bevacizumab after topical, subconjunctival, and intravitreal administration in rabbits. Investig. Ophthalmol. Vis. Sci..

[43] Sinapis C.I., Routsias J.G., Sinapis A.I., Sinapis D.I., Agrogiannis G.D., Pantopoulou A., Theocharis S.E., Baltatzis S., Patsouris E., Perrea D. (2011). Pharmacokinetics of intravitreal bevacizumab (Avastin^®^) in rabbits. Clin. Ophthalmol.

[44] Miyake T., Sawada O., Kakinoki M., Sawada T., Kawamura H., Ogasawara K., Ohji M. (2010). Pharmacokinetics of bevacizumab and its effect on vascular endothelial growth factor after intravitreal injection of bevacizumab in macaque eyes. Investig. Ophthalmol. Vis. Sci..

[45] Christoforidis J.B., Williams M.M., Kothandaraman S., Kumar K., Epitropoulos F.J., Knopp M.V. (2012). Pharmacokinetic properties of intravitreal I-124-aflibercept in a rabbit model using PET/CT. Curr. Eye Res..

[46] Dınc E., Yıldırım O., Necat Yılmaz S., Canacankatan N., Ayaz L., Ozcan T., Temel G.O. (2014). Intravitreal bevacizumab effects on VEGF levels in distant organs: An experimental study. Cutan. Ocul. Toxicol..

[47] Zhu Q., Ziemssen F., Henke-Fahle S., Tatar O., Szurman P., Aisenbrey S., Schneiderhan-Marra N., Xu X. (2008). Tübingen Bevacizumab Study Group; Grisanti, S. Vitreous levels of bevacizumab and vascular endothelial growth factor-A in patients with choroidal neovascularization. Ophthalmology.

[48] Yu L., Liang X.H., Ferrara N. (2011). Comparing protein VEGF inhibitors: In vitro biological studies. Biochem. Biophys. Res. Commun..

[49] Dedania V.S., Bakri S.J. (2016). Systemic safety of intravitreal anti-vascular endothelial growth factor agents in age-related macular degeneration. Curr. Opin. Ophthalmol..

[50] Moja L., Lucenteforte E., Kwag K.H., Bertele V., Campomori A., Chakravarthy U., D’Amico R., Dickersin K., Kodjikian L., Lindsley K. (2014). Systemic safety of bevacizumab versus ranibizumab for neovascular age-related macular degeneration. Cochrane Database Syst. Rev..

[51] Thulliez M., Angoulvant D., Le Lez M.L., Jonville-Bera A.-P., Pisella P.-J., Gueyffier F., Bejan-Angoulvant T. (2014). Cardiovascular events and bleeding risk associated with intravitreal antivascular endothelial growth factor monoclonal antibodies: Systematic review and meta-analysis. JAMA Ophthalmol..

[52] Krohne T.U., Eter N., Holz F.G., Meyer C.H. (2008). Intraocular pharmacokinetics of bevacizumab after a single intravitreal injection in humans. Am. J. Ophthalmol..

[53] Meyer C.H., Krohne T.U., Holz F.G. (2011). Intraocular pharmacokinetics after a single intravitreal injection of 1.5 mg versus 3.0 mg of bevacizumab in humans. Retina.

[54] Meyer C.H., Krohne T.U., Holz F.G. (2012). Concentrations of unbound bevacizumab in the aqueous of untreated fellow eyes after a single intravitreal injection in humans. Acta Ophthalmol..

[55] Park S.J., Choi Y., Na Y.M., Hong H.K., Park J.Y., Park K.H., Chung J.Y., Woo S.J. (2016). Intraocular Pharmacokinetics of Intravitreal Aflibercept (Eylea) in a Rabbit Model. Investig. Ophthalmol. Vis. Sci..

[56] Niwa Y., Kakinoki M., Sawada T., Wang X., Ohji M. (2015). Ranibizumab and Aflibercept: Intraocular Pharmacokinetics and Their Effects on Aqueous VEGF Level in Vitrectomized and Nonvitrectomized Macaque Eyes. Investig. Ophthalmol. Vis. Sci..

[57] Kaiser P.K., Kodjikian L., Korobelnik J.-F., Winkler J., Torri A., Zeitz O., Vitti R., Ahlers C., Zimmermann T., Dicioccio A.T. (2019). Systemic pharmacokinetic/pharmacodynamic analysis of intravitreal aflibercept injection in patients with retinal diseases. BMJ. Open Ophthalmol..

[58] Stewart M.W. (2014). Pharmacokinetics, pharmacodynamics and pre-clinical characteristics of ophthalmic drugs that bind VEGF. Expert Rev. Clin. Pharmacol.

[59] Do D.V., Rhoades W., Nguyen Q.D. (2019). Pharmacokinetic study of intravitreal aflibercept in humans with neovascular age-related macular degeneration. Retina.

[60] Christoforidis J.B., Williams M.M., Wang J., Jiang A., Pratt C., Abdel-Rasoul M., Hinkle G.H., Knopp M.V. (2013). Anatomic and pharmacokinetic properties of intravitreal bevacizumab and ranibizumab after vitrectomy and lensectomy. Retina.

[61] Christoforidis J.B., Xie Z., Jiang A., Wang J., Pratt C., Gemensky-Metzler A., Abdel-Rasoul M., Roy S., Liu Z. (2013). Serum levels of intravitreal bevacizumab after vitrectomy, lensectomy and non-surgical controls. Curr. Eye Res..

[62] Ahn J., Kim H., Woo S.J., Park J.H., Park S., Hwang D.J., Park K.H. (2013). Pharmacokinetics of intravitreally injected bevacizumab in vitrectomized eyes. J. Ocul. Pharmacol. Ther..

[63] Liu X., Ye J., Zhang Y., Liu Q., Bai R., Yuan W., Cai D., Zheng X., Bian Y., Zhou S. (2019). Ocular Biodistribution of ^89^Zr-Bevacizumab in New Zealand Rabbits Determined Using PET/MRI: A Feasibility Study. Iran. J. Radiol..

[64] Kakinoki M., Sawada O., Sawada T., Saishin Y., Kawamura H., Ohji M. (2012). Effect of vitrectomy on aqueous VEGF concentration and pharmacokinetics of bevacizumab in macaque monkeys. Investig. Ophthalmol. Vis. Sci..

[65] Missel P.J. (2012). Simulating intravitreal injections in anatomically accurate models for rabbit, monkey, and human eyes. Pharm. Res..

[66] Xu Q., Boylan N.J., Suk J.S., Wang Y.-Y., Nance E.A., Yang J.-C., McDonnell P.J., Cone R.A., Duh E.J., Hanes J. (2013). Nanoparticle diffusion in, and microrheology of, the bovine vitreous ex vivo. J. Control Release.

[67] Laude A., Tan L.E., Wilson C.G., Lascaratos G., Elashry M., Aslam T., Patton N., Dhillon B. (2010). Intravitreal therapy for neovascular age-related macular degeneration and inter-individual variations in vitreous pharmacokinetics. Prog. Retin. Eye Res..

[68] Angi M., Kalirai H., Coupland S.E., Damato B.E., Semeraro F., Romano M.R. (2012). Proteomic analyses of the vitreous humour. Mediat. Inflamm..

[69] Ulrich J.N., Spannagl M., Kampik A., Gandorfer A. (2008). Components of the fibrinolytic system in the vitreous body in patients with vitreoretinal disorders. Clin. Experiment. Ophthalmol..

[70] Del Amo E.M., Rimpelä A.-K., Heikkinen E., Kari O.K., Ramsay E., Lajunen T., Schmitt M., Pelkonen L., Bhattacharya M., Richardson D. (2017). Pharmacokinetic aspects of retinal drug delivery. Prog. Retin Eye Res..

[71] Gal-Or O., Dotan A., Dachbash M., Tal K., Nisgav Y., Weinberger D., Ehrlich R., Livnat T. (2016). Bevacizumab clearance through the iridocorneal angle following intravitreal injection in a rat model. Exp. Eye Res..

[72] Peters S., Heiduschka P., Julien S., Ziemssen F., Fietz H., Bartz-Schmidt K.U. (2007). Tübingen Bevacizumab Study Group; Schraermeyer, U. Ultrastructural findings in the primate eye after intravitreal injection of bevacizumab. Am. J. Ophthalmol..

[73] Heiduschka P., Fietz H., Hofmeister S., Schultheiss S., Mack A.F., Peters S., Ziemssen F., Niggemann B., Julien S., Bartz-Schmidt K.U. (2007). Penetration of bevacizumab through the retina after intravitreal injection in the monkey. Investig. Ophthalmol. Vis. Sci..

[74] Vellonen K.-S., Hellinen L., Mannermaa E., Ruponen M., Urtti A., Kidron H. (2018). Expression, activity and pharmacokinetic impact of ocular transporters. Adv. Drug Deliv. Rev..

[75] Krohne T.U., Muether P.S., Stratmann N.K., Holz F.G., Kirchhof B., Meyer C.H., Fauser S. (2015). Influence of ocular volume and lens status on pharmacokinetics and duration of action of intravitreal vascular endothelial growth factor inhibitors. Retina.

[76] Edington M., Connolly J., Chong N.V. (2017). Pharmacokinetics of intravitreal anti-VEGF drugs in vitrectomized versus non-vitrectomized eyes. Expert Opin Drug Metab Toxicol.

[77] Gisladottir S., Loftsson T., Stefansson E. (2009). Diffusion characteristics of vitreous humour and saline solution follow the Stokes Einstein equation. Graefes Arch. Clin. Exp. Ophthalmol..

[78] Stefánsson E. (2009). Physiology of vitreous surgery. Graefes Arch. Clin. Exp. Ophthalmol..

[79] Xu Y., You Y., Du W., Zhao C., Li J., Mao J., Chen H., Cheng L. (2012). Ocular pharmacokinetics of bevacizumab in vitrectomized eyes with silicone oil tamponade. Investig. Ophthalmol. Vis. Sci..

[80] Gadkar K., Pastuskovas C.V., Le Couter J.E., Elliott J.M., Zhang J., Lee C.V., Sanowar S., Fuh G., Kim H.S., Lombana T.N. (2015). Design and Pharmacokinetic Characterization of Novel Antibody Formats for Ocular Therapeutics. Investig. Ophthalmol. Vis. Sci..

[81] Lowe J., Maia M., Wakshull E., Siguenza P., Liu P., Lakhani S., Rusit J., Elliott R., Quarmby V. (2010). Development of a novel homogenous electrochemiluminescence assay for quantitation of ranibizumab in human serum. J. Pharm. Biomed. Anal..

[82] Zhang Y., Yao Z., Kaila N., Kuebler P., Visich J., Maia M., Tuomi L., Ehrlich J.S., Rubio R.G., Campochiaro P.A. (2014). Pharmacokinetics of ranibizumab after intravitreal administration in patients with retinal vein occlusion or diabetic macular edema. Ophthalmology.

[83] Lowe J., Wakshull E., Shek T., Chuntharapai A., Elliott R., Rusit J., Maia M. (2018). Development and validation of a novel semi-homogenous clinical assay for quantitation of Ranibizumab in human serum. J. Immunol. Methods.

[84] Dickmann L.J., Yip V., Li C., Abundes J., Maia M., Young C., Stainton S., Hass P.E., Joseph S.B., Prabhu S. (2015). Evaluation of Fluorophotometry to Assess the Vitreal Pharmacokinetics of Protein Therapeutics. Investig. Ophthalmol. Vis. Sci..

[85] Giannos S.A., Kraft E.R., Zhao Z.-Y., Merkley K.H., Cai J. (2018). Formulation Stabilization and Disaggregation of Bevacizumab, Ranibizumab and Aflibercept in Dilute Solutions. Pharm. Res..

[86] Muether P.S., Hermann M.M., Dröge K., Kirchhof B., Fauser S. (2013). Long-term stability of vascular endothelial growth factor suppression time under ranibizumab treatment in age-related macular degeneration. Am. J. Ophthalmol..

[87] Saunders D.J., Muether P.S., Fauser S. (2015). A model of the ocular pharmacokinetics involved in the therapy of neovascular age-related macular degeneration with ranibizumab. Br. J. Ophthalmol..

[88] Celik N., Scheuerle A., Auffarth G.U., Kopitz J., Dithmar S. (2015). Intraocular Pharmacokinetics of Aflibercept and Vascular Endothelial Growth Factor-A. Investig. Ophthalmol. Vis. Sci..

[89] Mould D.R., Upton R.N. (2012). Basic concepts in population modeling, simulation, and model-based drug development. CPT Pharmacomet. Syst Pharmacol.

[90] Sheiner L.B., Rosenberg B., Melmon K.L. (1972). Modelling of individual pharmacokinetics for computer-aided drug dosage. Comput. Biomed. Res..

[91] Sheiner L.B., Beal S.L. (1980). Evaluation of methods for estimating population pharmacokinetics parameters. I. Michaelis-Menten model: Routine clinical pharmacokinetic data. J. Pharmacokinet. Biopharm..

[92] Stanski D.R., Maitre P.O. (1990). Population pharmacokinetics and pharmacodynamics of thiopental: The effect of age revisited. Anesthesiology.

[93] Manolis E., Brogren J., Cole S., Hay J.L., Nordmark A., Karlsson K.E., Lentz F., Benda N., Wangorsch G., Pons G. (2017). Commentary on the MID3 Good Practices Paper. CPT Pharmacomet. Syst. Pharmacol..

[94] Marshall S., Burghaus R., Cosson V., Cheung S., Chenel M., DellaPasqua O., Frey N., Hamrén B., Harnisch L., Ivanow F. (2016). Good Practices in Model-Informed Drug Discovery and Development: Practice, Application, and Documentation. CPT Pharmacomet. Syst. Pharmacol..

